# Survival, bacterial clearance and thrombocytopenia are improved in polymicrobial sepsis by targeting nuclear transport shuttles

**DOI:** 10.1371/journal.pone.0179468

**Published:** 2017-06-19

**Authors:** Ruth Ann Veach, Yan Liu, Jozef Zienkiewicz, Lukasz S. Wylezinski, Kelli L. Boyd, James L. Wynn, Jacek Hawiger

**Affiliations:** 1 Immunotherapy Program at Vanderbilt University School of Medicine, Nashville, Tennessee, United States of America; 2 Department of Medicine, Division of Allergy, Pulmonary and Critical Care Medicine, Vanderbilt University School of Medicine, Nashville, Tennessee, United States of America; 3 Department of Veterans Affairs, Tennessee Valley Health Care System, Nashville, Tennessee, United States of America; 4 Department of Molecular Physiology and Biophysics, Vanderbilt University School of Medicine, Nashville, Tennessee, United States of America; 5 Department of Pathology, Microbiology and Immunology, Vanderbilt University School of Medicine, Nashville, Tennessee, United States of America; 6 Department of Pediatrics, University of Florida, Gainesville, Florida, United States of America; 7 Department of Pathology, Immunology, and Laboratory Medicine, University of Florida, Gainesville, Florida, United States of America; National Institutes of Health, UNITED STATES

## Abstract

The rising tide of sepsis, a leading cause of death in the US and globally, is not adequately controlled by current antimicrobial therapies and supportive measures, thereby requiring new adjunctive treatments. Severe microvascular injury and multiple organ failure in sepsis are attributed to a “genomic storm” resulting from changes in microbial and host genomes encoding virulence factors and endogenous inflammatory mediators, respectively. This storm is mediated by stress-responsive transcription factors that are ferried to the nucleus by nuclear transport shuttles importins/karyopherins. We studied the impact of simultaneously targeting two of these shuttles, importin alpha 5 (Imp α5) and importin beta 1 (Imp β1), with a cell-penetrating Nuclear Transport Modifier (NTM) in a mouse model of polymicrobial sepsis. NTM reduced nuclear import of stress-responsive transcription factors nuclear factor kappa B, signal transducer and activator of transcription 1 alpha, and activator protein 1 in liver, which was also protected from sepsis-associated metabolic changes. Strikingly, NTM without antimicrobial therapy improved bacterial clearance in blood, spleen, and lungs, wherein a 700-fold reduction in bacterial burden was achieved while production of proinflammatory cytokines and chemokines in blood plasma was suppressed. Furthermore, NTM significantly improved thrombocytopenia, a prominent sign of microvascular injury in sepsis, inhibited neutrophil infiltration in the liver, decreased L-selectin, and normalized plasma levels of E-selectin and P-selectin, indicating reduced microvascular injury. Importantly, NTM combined with antimicrobial therapy extended the median time to death from 42 to 83 hours and increased survival from 30% to 55% (*p* = 0.022) as compared to antimicrobial therapy alone. This study documents the fundamental role of nuclear signaling mediated by Imp α5 and Imp β1 in the mechanism of polymicrobial sepsis and highlights the potential for targeting nuclear transport as an adjunctive therapy in sepsis management.

## Introduction

**Fig 1:**
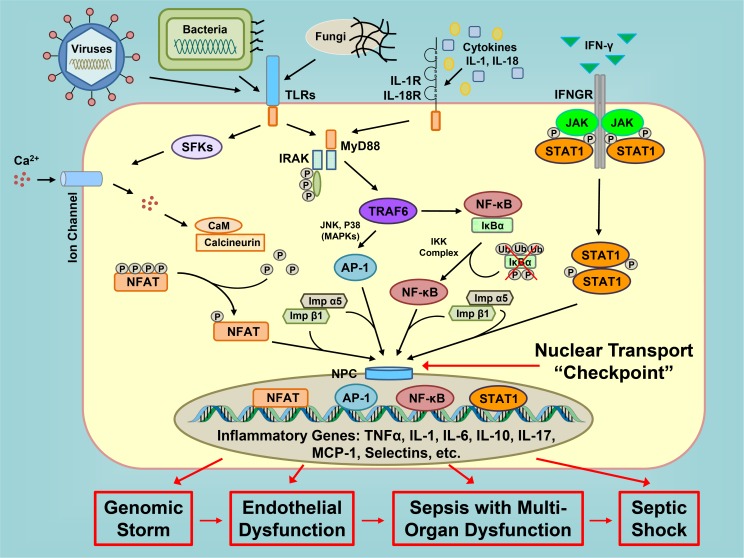
Nuclear transport is a pivotal checkpoint in genomic regulation of microbial inflammation. Inflammatory signaling cascades initiated by cell responses to microbial virulence factors and cytokines culminate in nuclear translocation of stress-responsive transcription factors (SRTFs) that upregulate inflammatory gene networks. Unchecked, this genomic reprogramming (genomic storm) leads to endothelial dysfunction, multi-organ failure and ultimately fatal shock. Inhibiting nuclear transport at a common “checkpoint” located downstream of TLRs and cytokine receptors globally suppresses expression of inflammatory genes thereby calming the genomic storm and averting multiple organ injury. Legend: TLRs (Toll-like receptors); Ca^2+^ (calcium ions); IL-1R and IL-18R (interleukin 1 and 18 receptors, respectively); IFN-γ (Interferon-gamma); IFNGR (Interferon-gamma receptor); SFKs (Src family of protein tyrosine kinases); MyD88 (Myeloid Differentiation Primary Response 88); IRAK (interleukin-1 receptor-associated kinase); JAK (Janus kinase); P (Phosphate group); Ub (Ubiquitin); CaM (Calmodulin); TRAF6 (TNF receptor-associated factor 6); IKK (I kappa B kinase); IκBα (NF-κB inhibitor alpha); JNK (c-Jun N-terminal kinase); MAPKs (mitogen-activated protein kinases); NFAT (nuclear factor of activated T cells); AP-1 (Activator protein 1); NF-κB (Nuclear factor kappa B); NPC (nuclear pore complex); STAT1 (signal transducer and activator of transcription 1); Imp α5 (Importin alpha 5); Imp β1 (Importin beta 1); TNFα (tumor necrosis factor alpha); IL-1, IL-6, IL-10 and IL-17 (interleukin 1, 6, 10 and 17, respectively); MCP-1 (Monocyte Chemoattractant Protein-1).

Sepsis, a complication of systemic or localized infections due to bacterial, fungal or viral pathogens, represents one of the most challenging problems to prevent and treat in modern hospitals [1]. Serious intravascular or extravascular infections culminate in severe microvascular endothelial injury that underlies hypotension and is difficult to reverse despite initiation of antimicrobial therapy and supportive measures. Microbial agents that escape defenses mounted by the innate and adaptive immune systems are not fully controlled by specific antimicrobial therapy, which is often delayed [2]. During their uncontrolled growth, especially in extravascular loci, bacteria reprogram their genomes through a quorum sensing mechanism and produce multiple virulence factors that potentiate microvascular injury [3–5]. The host’s innate immune responses are initiated by bacterial and fungal virulence factors and viral nucleic acids through the pattern recognition receptors, *e*.*g*. Toll-like receptors, present in immune cells and non-immune endothelial and epithelial cells [6,7]. Signals produced by these receptors are relayed to the cell’s nucleus through activation of stress responsive transcription factors (SRTFs), such as nuclear factor kappa B (NF-κB), activator protein 1 (AP-1), nuclear factor of activated T-cells (NFAT), and signal transducer and activator of transcription 1 alpha (STAT-1α) [8]. Each of these transcription factors, either alone or in combination, triggers expression of multiple genes that encode proinflammatory cytokines and chemokines, as well as their receptors, signal transducers, and cell adhesion molecules, a response termed a genomic storm [9,10]. The products of this genomic reprogramming mediate fever, microvascular endothelial instability responsible for low blood pressure, microvascular endothelial injury that underlies acute respiratory distress syndrome, disseminated intravascular coagulation, and multiple organ dysfunction, culminating in potentially lethal vascular collapse refractory to vasopressors and fluid resuscitation, a condition known as septic shock [11,12]. Thus, reprograming of gene regulatory networks in response to a multitude of microbial insults is dependent on signaling to the host cell’s nucleus comprising a fundamental process of microbial inflammation (see Fig 1 for a conceptual depiction).

Small transcription factors (<50 kD), usually those regulating the housekeeping genes that encode cell survival factors, have free passage from the cytoplasm to the nucleus [13]. In contrast, nuclear transport of transcription factors larger than 50 kD, such as SRTFs, is guided by one or more nuclear localization sequences (NLSs). These intracellular “zip codes” are displayed on SRTFs upon stimulation of immune and non-immune cells by microbial insults. NLSs are then recognized by nuclear transport adaptor proteins, importins/karyopherins alpha (Imp α) (see Fig 1). The stimulus-induced formation of SRTF and importins α complexes also encompasses importin beta 1 (Imp β1), which is recognized by nuclear pore proteins to allow translocation of the cargo to the nucleus [8,14]. Until recently, nuclear transport has been targeted through the forced expression of genes that encode inhibitors of proinflammatory SRTFs, such as the degradation-resistant inhibitor of NF-κB termed IκBα [15,16]. However, NF-κB is only one of multiple SRTFs that mediate signaling to the nucleus in response to infection. Other SRTFs, such as AP-1, STAT1 and NFAT, are also transported to the nucleus during the inflammatory response yet their nuclear transport is not impeded by IκBα; contrarily, the AP-1 pathway is activated [17]. We hypothesized that targeting nuclear transport, a pivotal checkpoint integrating translocation of multiple transcription factors to the nucleus, would be a more efficient strategy than targeting signaling pathways of individual transcription factors. This concept was proven by design and development of Nuclear Transport Modifiers (NTMs). NTMs target the nuclear transport shuttles, Imp α5 and Imp β1, that translocate SRTFs to the nucleus and control signal transduction pathways, which culminate in genomic reprogramming [1,8,10]. In recent preclinical studies, we used a highly soluble cell-penetrating NTM (cSN50.1), with dual specificity. This NTM has segments that bind both Imp α5, which recognizes NLS derived from NFκB1 [18], and Imp β1, which recognizes the signal-sequence hydrophobic region (SSHR) derived from Fibroblast Growth Factor 4 [19]. SSHR also serves as a membrane translocating motif (MTM) to enable intracellular delivery of peptides and proteins through an ATP-and endocytosis-independent mechanism [20]. This and other NTMs have been shown to inhibit nuclear translocation of SRTFs [21] and thereby reduce inflammatory responses, microvascular injury, apoptosis and hemorrhagic necrosis with a concomitant gain in survival, in models of lethal shock induced by bacterial toxins [22–24].

Now, we report that in an experimental model of polymicrobial sepsis, simultaneous targeting of nuclear transport adaptors Imp α5 and Imp β1 with cSN50.1 attenuated nuclear import of SRTFs and maintained metabolically-important glycogen stores in the liver. NTM treatment led to a significantly reduced bacterial burden in blood, spleen, and lungs, attenuated proinflammatory cytokines and chemokines in blood, preserved normal platelet count in blood, and reduced plasma markers of microvascular injury as well as neutrophil infiltration of the liver. Moreover, combining NTM with antibiotic therapy significantly extended mean time to death, and almost doubled survival.

## Results

### NTM attenuates nuclear transport of SRTFs in liver cells and reduces bacterial burden in blood, spleen, and lungs of septic mice

As Gram-negative bacteria are the causes of sepsis in two-thirds of patients either alone or in combination with other microbes [25], we employed a clinically relevant model of polymicrobial sepsis. In this non-surgical model, peritonitis is induced by standardized challenge with bacteria–rich gut microbiome. Freshly obtained gut microbiome, in the form of a cecal slurry (CS), was injected intraperitoneally thereby avoiding surgical wounding and uncontrolled spillage of cecal contents into the peritoneal cavity inherent to surgical cecal ligation and puncture [26]. We tested the hypothesis that controlling nuclear import of SRTFs with cSN50.1, the newest, highly soluble cell-penetrating NTM peptide [10], would reduce production of sepsis-associated mediators of microvascular injury, improve the clearance of bacteria in blood and major organs, and increase the effectiveness of antimicrobial therapy thereby improving survival.

**Fig 2:**
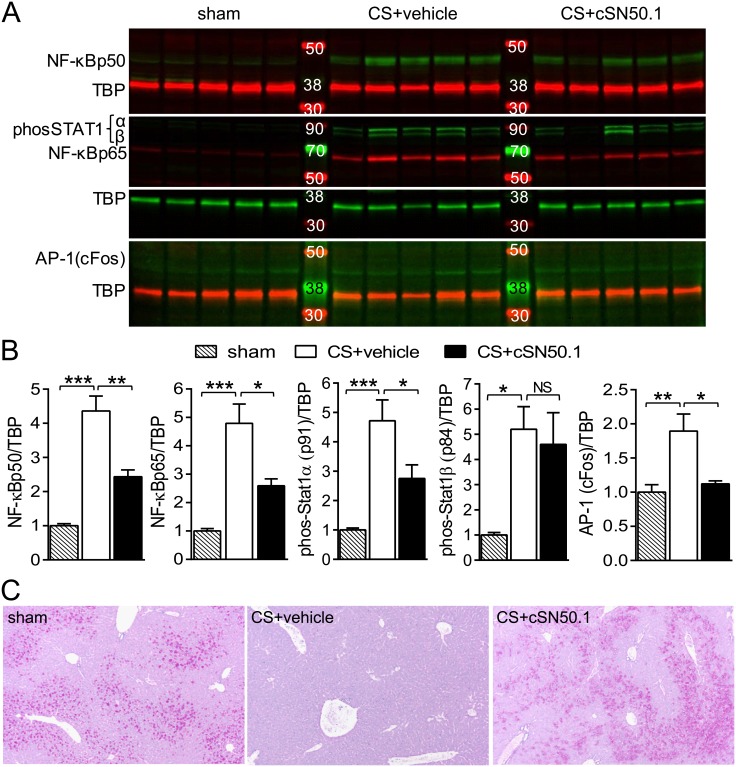
Nuclear transport of SRTFs is reduced and normal liver architecture is protected in infected mice treated with NTM. (**A**) Nuclear content of NF-κBp50, NF-κBp65, pY701 phospho-STAT1α (MW 91) and -STAT1β (MW 84), and AP-1 (cFos) in livers collected 12 h after infection from sham-infected or CS-infected mice treated with NTM (cSN50.1) or vehicle. Each lane is the nuclear extract from one mouse; lanes shown in each row are from the same gel. Immunoblots shown are representative of 2 independent nuclear extract preparations. (**B**) Quantitative analysis of immunoblots shown in **A.** All samples were normalized to TATA binding protein (TBP) on the same immunoblot (Data points are displayed as mean + SEM of fold change, *n* = 10/group; *p < 0.05, **p<0.005, ***p<0.0001 by *t* test, NS = not significant). (**C**) Liver sections collected 12 h after infection from sham-infected or CS-infected mice treated with NTM (cSN50.1) or vehicle stained with PAS. Images are representative of 5 mice/group.

Patients who succumb to sepsis display strikingly increased levels of a key SRTF, NF-κB, in nuclei of peripheral blood mononuclear cells [15]. Consistent with this clinical hallmark, we found not only increased nuclear content of NF-κB1 (p50) and NF-κB RelA (p65) but also phosphorylated STAT1α (MW91), phosphorylated STAT1β (MW84) and AP-1 (cFos) in liver cells of septic animals comprising hepatocytes, macrophages, and microvascular endothelial cells, among other cells (Fig 2A). Treatment with NTM significantly reduced the nuclear content of NF-κB1 (p50) and NF-κB RelA (p65), phosphorylated STAT1α and AP-1 (cFos) (Fig 2B). STAT1β was not reduced by NTM treatment. Though tyrosine phosphorylation of both STAT1α and β is induced by the inflammatory cytokine IFN-γ, STAT1β is a transcriptionally inactive truncated isoform and acts as a dominant-negative inhibitor of STAT1α [27]. Proinflammatory signaling to the nucleus has also been implicated in rapid glycogen depletion in the liver during sepsis [28]. Accordingly, NTM treatment prevented glycogenolysis in the livers of infected mice, demonstrated by Periodic Acid-Schiff stain (PAS) (Fig 2C). In earlier studies of lipopolysaccharide toxemia, NTM also prevented glycogen depletion from liver [23].

**Fig 3:**
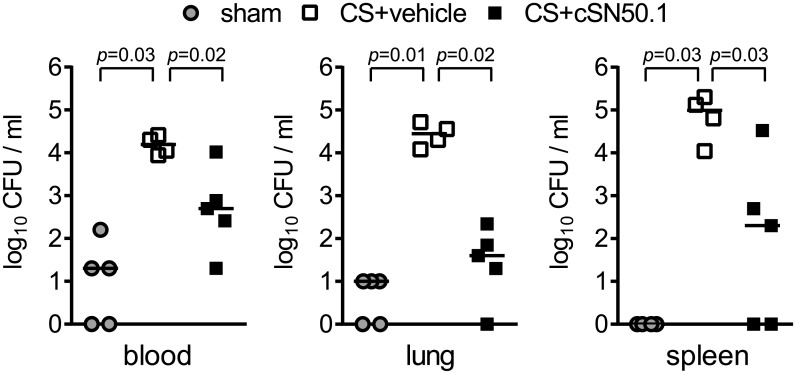
Bacterial clearance is improved in infected mice treated with NTM. CFU determined by serial dilution of whole blood and organ homogenates collected 12 h after infection from sham- or CS-infected mice treated with NTM (cSN50.1) or vehicle. Bars represent median values from 4–5 mice/group (*p* values determined by Mann-Whitney test).

Efficient microbial clearance is essential for survival from polymicrobial sepsis [29]. Remarkably, in the absence of antimicrobial therapy, CS-infected mice treated with NTM- displayed enhanced bacterial clearance from blood and target organs (spleen and lung) by 12 h after infection (Fig 3). A direct antimicrobial effect of NTM can be excluded as cSN50.1 peptide did not inhibit the growth of cecal bacteria tested *ex vivo* (data not shown). The most dramatic reduction in bacterial load (about 700-fold) was noted in the lungs while bacteria in spleen and blood were also significantly reduced. Negligible detection of bacteria in sham-infected animals can be attributed to multiple intraperitoneal injections and repeated blood collections. These results indicate that the function of blood-borne and tissue phagocytes in regard to bacterial clearance is preserved, or even enhanced, when proinflammatory signaling to the nucleus is reduced in NTM-treated infected animals.

### NTM attenuates inflammatory mediators and their downstream effects on immune signaling

**Fig 4:**
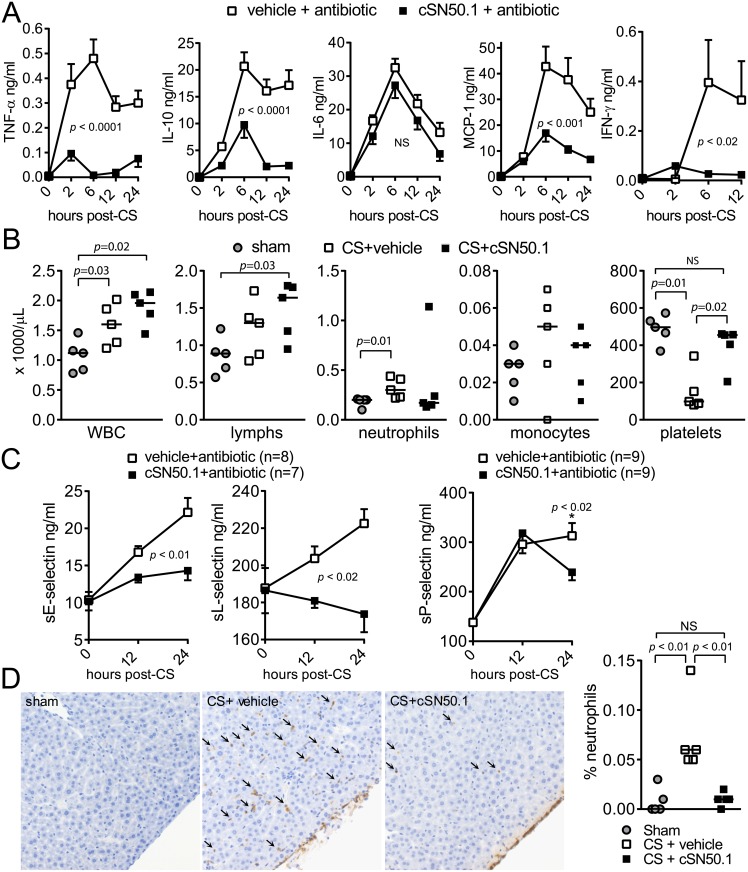
NTM modulates blood and vascular responses to sepsis. (**A**) Cytokines/chemokine measured in blood plasma by cytometric bead array before and at 2 h, 6 h, 12 h, and 24 h after CS infection. Antibiotic therapy with meropenem began at 12 h post-infection. (*n* = 20 mice/group; *p* values calculated by two-way repeated measures ANOVA). IFN-γ measured by ELISA in blood plasma before and at 2 h, 6 h and 12 h after CS infection. (vehicle + antibiotic, *n* = 8; cSN50.1 + antibiotic, *n* = 13; *p* values calculated by two-way repeated measures ANOVA). (**B**) Total WBC, lymphocytes, neutrophils, monocytes, and platelets in whole blood collected 12 h after infection from sham-infected or CS-infected mice treated with NTM (cSN50.1) or vehicle. Bars represent median values from 5 mice/group (*p* values determined by Mann-Whitney test). (**C**) Soluble E-selectin, L-selectin, and P-selectin measured in blood plasma before and at 12 h and 24 h after CS infection. Antibiotic therapy with meropenem began at 12 h post-infection. (*p* values for sE-selectin and sL-selectin calculated by two-way repeated measures ANOVA, *p* value for sP-selectin determined by Mann-Whitney test at 24 h only, n = 9/group). (**D**) Representative images (400x magnification) and quantification of neutrophils (indicated by arrows) in the hepatic parenchyma of livers collected 12 h after infection from sham-infected or CS-infected mice treated with NTM (cSN50.1) or vehicle. The percentage of positive brown (DAB-positive) stained cells was calculated as a total analyzed number of positive cells divided by total number cells in the section of liver. Bars represent median values from 5 mice/group (*p* values determined by Mann-Whitney test).

During the early hyperinflammatory phase of sepsis, plasma levels of proinflammatory cytokines TNF-α and IL-6, as well as the chemokine monocyte chemoattractant protein 1 (MCP-1/CCL2), are dramatically increased. In concert, the type II interferon, interferon gamma (IFN-γ), enhances cell-mediated immune responses. IFN-γ is a specific activator of macrophage function and plays a regulatory role in the host immune defense to bacterial infection. Remarkably, administration of anti-IFN-γ antibody significantly decreased bacterial load in the peritoneum in a rat model of peritonitis [30]. Reduced expression of vascular endothelial (VE) cadherin, the mainstay of microvascular integrity, was observed in IFN-γ-stimulated human pulmonary microvascular endothelial cells and in the endothelium of all vessels obtained from patients with Gram-negative bacterial sepsis complicated by ARDS [31]. Production of anti-inflammatory cytokines, such as IL-10, is also triggered to restore inflammatory homeostasis. However, if excessive production of inflammatory mediators prevails, the consequences are vascular dysfunction, multi- organ failure and death [29]. The attenuation of nuclear signaling mediated by SRTFs was accompanied by significant suppression of plasma levels of TNF-α, MCP-1, and IFN-γ, whereas the pattern of IL-6 response remained unchanged (Fig 4A). While increased IL-10 provides a feedback mechanism to suppress TNF-α expression and counteract excessive immune responses, overproduction of IL-10 reduces bacterial clearance [32]. Continued overexpression of IL-10 contributes to the immunosuppressive environment that characterizes later stages of sepsis [29]. Cumulatively, the sepsis-induced proinflammatory response, with the notable exception of IL-6, is suppressed by NTM, contributing to maintaining homeostatic regulation.

Consistent with the role of phagocytes in bacterial clearance from blood and organs, both vehicle and cSN50.1-treated animals displayed a significant increase in total circulating leukocytes compared to sham-infected animals at 12 hours after sepsis was initiated, indicating that lymphocyte, neutrophil and monocyte counts were not suppressed by treatment with NTM (cSN50.1) (Fig 4B). Strikingly, NTM preserved platelet counts (Fig 4B) while thrombocytopenia, manifested by a drop in platelet count in blood, was evident in vehicle-treated mice. This hallmark of severe bacterial infection is a marker of poor outcome, presumably due to platelet entrapment by injured microvascular surfaces and subsequent formation of platelet thrombi [33]. Acute platelet consumption and microvascular dysfunction depend on induction of genomic reprogramming regulated by SRTFs and controlled by their nuclear import [23]. Other parameters of the complete blood count were not significantly altered by infection or NTM treatment (S1 Fig).

Selectins play a critical role in inflammation by regulating tethering and rolling of neutrophils along the microvascular endothelium, a prerequisite for subsequent adhesion and emigration. Leukocyte migration appears to be the initial event that leads to multiple organ inflammation and dysfunction during sepsis. Interfering with selectin-mediated rolling of leukocytes prevents their extravascular migration and subsequent organ dysfunction [34,35]. L-selectin is constitutively expressed on leukocytes and is rapidly released to the bloodstream in response to activation [36,37]. E-selectin is not stored in endothelial cells; rather it is expressed due to genomic upregulation evoked by lipopolysaccharide, the primary virulence factor of Gram-negative bacteria, and by cytokines, such as TNF-α. E-selectin is then released into the bloodstream [38,39]. P-selectin is expressed by activated platelets and endothelial cells, mediating platelet—leukocyte and platelet—endothelial cell interactions and contributing to thrombi formation [40]. NTM, in addition to antibiotic treatment, attenuated expression of soluble P-selectin in blood plasma and prevented elevation of soluble E-selectin, while plasma levels of these markers of endothelial damage continued to rise with antibiotic treatment alone (Fig 4C).

Consistent with reduced cytokine, chemokine and selectins production, NTM treatment inhibited liver infiltration by neutrophils (Fig 4D). Migration of neutrophils into the hepatic parenchyma was not observed in the sham-infected animals. In infected mice treated with vehicle, increased numbers of neutrophils were observed in the hepatic parenchyma, indicating systemic inflammation. In contrast, CS-infected mice treated with NTM displayed neutrophils aggregated mostly on the serosal surface with a few cells in the hepatic parenchyma, suggesting localized inflammation in the peritoneal cavity following the injection of gut microbiome.

Cumulatively, these parameters of microvascular inflammation were significantly suppressed when infected mice were treated with NTM either alone or in combination with antimicrobial therapy, further indicating the overall dependence of this process on the nuclear transport of SRTFs.

### Nuclear signaling modulation in combination with antimicrobial therapy increases survival in sepsis

**Fig 5:**
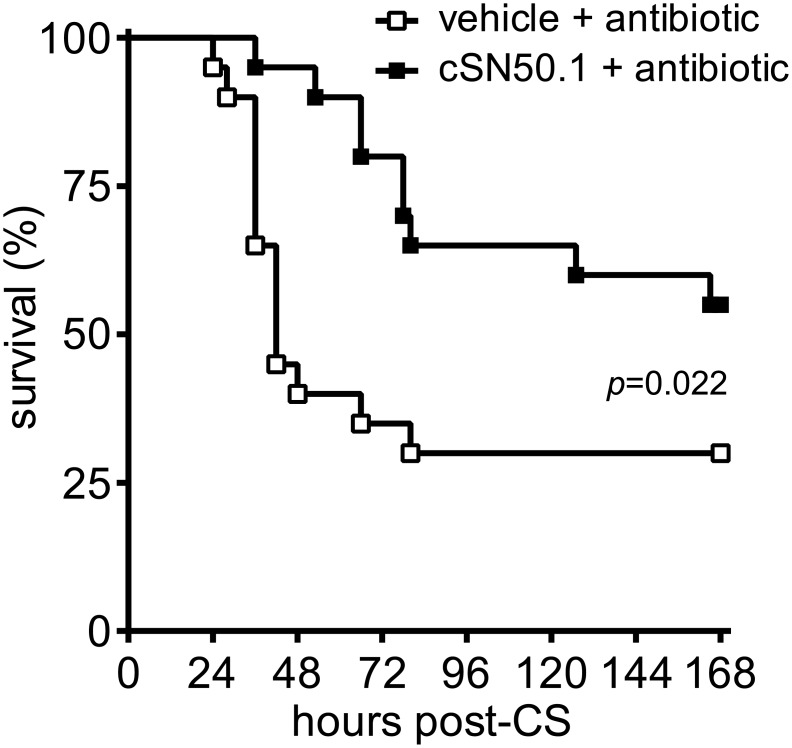
Survival is increased by combining NTM treatment with antibiotic therapy. Mice were infected with CS and treated with vehicle or NTM (cSN50.1), both supplemented by antibiotic therapy with meropenem (*n* = 20 mice/group; Kaplan-Meier survival plot with *p* value calculated by log rank analysis).

We next asked whether the beneficial actions of NTM in the acute phase of polymicrobial sepsis would exert a positive effect on overall survival in sepsis. To test this hypothesis, we combined NTM treatment with antimicrobial therapy in a model of polymicrobial sepsis. As documented in Fig 5, survival among animals treated only with meropenem, a broad-spectrum β-lactam antibiotic commonly used to treat severe infections, was 30% with median survival at 42 h. In contrast, when meropenem treatment was combined with NTM, survival was significantly increased to 55% and mean time to death was extended to 83 h (Fig 5). This gain in survival indicates that targeting proinflammatory nuclear signaling with NTM prevents irreversible multiple organ injury in sepsis when runaway infection cannot be controlled by antimicrobial therapy alone.

## Discussion

This study demonstrates that the dynamics and outcome of polymicrobial sepsis in a clinically relevant experimental model can be improved by targeting nuclear transport of stress-responsive transcription factors responsible for the genomic storm triggered by microbial virulence factors and endogenous mediators. Sepsis-induced signaling to the nucleus is responsible for blood and vascular system responses that contribute to multiple organ failure [1]. Mechanistically, NTM penetrates the plasma membrane of several cell types involved in sepsis and targets Imp α5 and Imp β1 responsible for nuclear signaling mediated by the translocation of SRTFs from the cytoplasm to the nucleus. The nuclear transport of SRTFs is an attractive target for therapeutic intervention, as selective targeting of Imp α5 and Imp β1 by the NTM, cSN50.1 peptide, suppresses activation of a myriad of proinflammatory and metabolic genes. By impeding nuclear import of NFκB and other SRTFs (AP-1 and STAT1), NTM suppressed expression of proinflammatory mediators including cytokines, chemokines, and soluble forms of cell adhesion molecules (selectins). Notably, NTM treatment in the absence of antimicrobial therapy enhanced clearance of multiple microbial species derived from the gut microbiome. This was especially notable in lungs wherein a 700-fold reduction in bacterial burden was obtained. Such an effect of NTM treatment could be linked to early suppression of IL-10, which has been demonstrated to impair the phagocytic function of alveolar macrophages [32]. This action of NTM is consistent with a previous study in which modulating nuclear transport in a model of rapidly progressing pulmonary anthrax also improved bacterial clearance from lungs while antimicrobial therapy alone was insufficient [41]. Thus, NTM-enhanced bacterial clearance indicates that this mode of targeting nuclear transport is not detrimental to the function of phagocytic cells in lungs and other organs, or to blood bactericidal activity. Of note, cell type-specific gene ablation of Toll-like receptor-4 (TLR4) in hepatocytes led to enhanced macrophage phagocytosis, lower bacterial levels, and improved survival in a model of polymicrobial sepsis caused by cecal ligation and puncture without antibiotic therapy [42]. Thus, NTM targeting of nuclear transport achieves a similar outcome as genetic ablation of TLR4 in terms of bacterial clearance.

TLR4 is a canonical receptor for LPS, a virulence factor known as the most active biologic inducer of systemic inflammation [43]. LPS expressed by Gram-negative bacteria is highly diverse, displaying striking structural diversity and corresponding biologic activity, as exemplified by *Escherichia coli* and *Neisseria meningitides* [44]. Analysis of systemic inflammation induced by LPS derived from a single bacterial species provides valuable information about the mechanism of action of that particular LPS. However, a polymicrobial sepsis model such as the one used in this study, which is based on intraperitoneal injection of gut microbiome (“cecal slurry”), infects the host with a multitude of Gram-negative bacteria expressing diverse LPS structures and additional virulence factors as well as Gram-positive bacteria and other microbes. Therefore, this clinically-relevant model raises the stakes for the host’s defenses and poses a greater challenge for testing new and more effective countermeasures.

In this complex setting of polymicrobial sepsis, targeting of the nuclear transport checkpoint (see Fig 1) offers a conceptual advantage. This checkpoint controls the traffic of stress-responsive transcription factors that display NLSs, recognized and ferried by nuclear transport shuttles from the cytoplasm to the nucleus. A family of six importins α are the mainstays of nuclear transport in mammalian cells [18]. In resting, unstimulated cells, importins α cannot recognize NLS motifs in SRTFs due to their shielding, *e*.*g*. by inhibitor of NF-κB (IκBα) or by posttranslational modifications, *e*.*g*. phosphorylation of NFAT [8] (see Fig 1). Following cell activation-induced degradation of IκBα and/or other modifications (e.g. dephosphorylation), NLS motifs are exposed on SRTFs, allowing them to complex with importins α and β for nuclear translocation. Once inside the nucleus, free SRTFs bind to their cognate sites in DNA and initiate gene transcription, leading to genome-wide reprogramming. Thus, the transition of immune and non-immune cells from a resting to an activated state is controlled by the nuclear translocation of transcription factors to the nucleus [8]. During this process, SRTFs activate a plethora of genes that encode inflammatory cytokines and chemokines, signal transducers (cyclooxygenase, nitric oxide synthase), and cell adhesion molecules, a truly massive response known as a genomic storm [9,10].

In turn, multiple signaling networks of cytokines and chemokines contribute to the mechanism of adult and neonatal sepsis by aggravating microvascular injury initiated by microbial agents that ignite a genomic storm [1,45]. Notably, NTM alone improves bacterial clearance and hematologic indices of polymicrobial adult sepsis but is not sufficient to improve survival without concomitant antimicrobial therapy. Conversely, antimicrobial therapy alone is not sufficient to reduce lethality in an experimental anthrax sepsis model [41]. Thus, combination of antimicrobial therapy and normalization of sepsis-induced dysregulation of gene regulatory networks by NTM led to significant improvement in survival in two distinct experimental sepsis models based on airway and peritoneal routes of infection.

In most studies of clinical sepsis in adult patients, increased levels of selectins are indicators of sepsis, with higher levels generally indicating increased severity of disease and mortality. However, in some studies, survival has been correlated with higher plasma levels of soluble selectins. It is postulated that the lower levels of soluble adhesion molecules in patients with worse outcomes are due to disrupted shedding, with elevated levels of cell surface adhesion molecules retained on the cell surface [46]. However, those studies evaluated sL- and sE-selectins in the context of rampant infection, not infection where inflammatory signaling has been ameliorated. Indeed, selectins are necessary for effective neutrophil recruitment [47]. In our experiments, lower soluble levels of sE- and sP-selectins in NTM-treated mice indicate reduced endothelial damage due to ameliorated inflammatory signaling, not aberrant shedding. This is also supported by lower levels of sL-selectin and decreased leukocyte extravasation into livers of NTM-treated animals. Additionally, increased bacterial clearance in NTM-treated animals precludes the need for increased extravasation to fight infection. Therefore our results are not contradictory, but instead indicate that reduced inflammatory signaling due to inhibited nuclear translocation prevents activation of the cells that shed selectins. Thus, the complex modulation of several SRTFs by NTM leads to normalized signaling through multiple inflammatory signaling pathways, not just an effect on any one of them (see Fig 1).

This study of nuclear transport signaling in the mechanism of polymicrobial sepsis opens up a new avenue for exploring innovative approaches to restore the complex balance of pro- and anti-inflammatory mechanisms in blood and vascular systems that would allow for successful recovery from sepsis. However, further studies are needed to examine the mechanisms at play in resolving sepsis. The discovery of lipid mediators, such as resolvin D1, that regulate inflammation, reduce apoptosis of lymphocytes, and promote a return to homeostasis is one of many emerging approaches in sepsis research [48]. Notably, resolvin D1 inhibits ER stress-induced apoptosis of liver cells by reducing sterol regulatory element-binding protein (SREBP) 1 expression and caspase 3 activity [49]. As recently demonstrated in a model of hyperlipidemia, the bi-functional NTM cSN50.1 used herein also modulates nuclear transport of SREBPs and carbohydrate-responsive element binding protein (ChREPB), transcription factors responsible for lipid and carbohydrate homeostasis, respectively, in addition to reducing nuclear transport of SRTFs [19]. Thus, NTM-regulated expression and action of SREBPs and ChREBP [19], as well as reduction of endotoxin-induced caspase 3/7 activity [23], offer potential mechanisms for suppression of glycogenolysis in early sepsis and cytoprotective effects in the later, immunosuppressive stage of sepsis. A study characterizing metabolomic, proteomic and clinical variables in sepsis survival and death delineated robust and reproducible differences in host responses to sepsis in survivors and nonsurvivors. Glycolysis, gluconeogenesis and the citric acid cycle differed prominently between survivors and nonsurvivors of community-acquired sepsis, and accumulation of carnitine esters with medium- or short-chain fatty acids and branched-chain amino acids were the most pronounced biochemical markers identified in nonsurvivors [50]. How NTM may enhance resolution of sepsis through its known corrective effect on metabolic signaling [19] in concert with its anti-inflammatory properties offers an intriguing prospect for future studies.

Taken together, our experimental study identifies nuclear transport as a key step in the development of irreversible microvascular injury that contributes to the lethal outcome of polymicrobial sepsis. Targeting the nuclear transport of proinflammatory transcription factors, as demonstrated in this study, and metabolic transactivators documented elsewhere [19], attenuates the initiation and progression of microbial and metabolic inflammation that underlie sepsis. Further analysis of the crosstalk between these signaling pathways in sepsis may increase a better understanding of its mechanism and resolution.

## Materials and methods

### Peptide synthesis and purification

Highly soluble cell-penetrating NTM peptide cSN50.1, was synthesized, purified, and filter-sterilized as described elsewhere [10,18,19].

### Animals, infection and sample collection

Eight week-old female C57Bl/6J mice (Jackson Laboratories) were used in all experiments and randomly assigned to treatment groups. For survival and blood cytokine/chemokine analyses, all mice were injected i.p. with 2 mg CS/g body weight (5.5x10^8^CFU/kg). CS was freshly prepared for each experiment as described by Wynn et al [26]. Briefly, healthy 6 week-old female mice were euthanized less than 2 weeks after arrival from the vendor, their cecum isolated and the cecal contents (“gut microbiome”) extruded, weighed, and suspended in 5% dextrose at a concentration of 80 mg/ml. NTM peptide cSN50.1 was dissolved in sterile water and diluted with sterile physiological saline to a final concentration of 3.3 mg/ml in 0.45% w/v of NaCl. Mice were given NTM (cSN50.1 peptide, 0.66 mg/injection), or vehicle (0.45% w/v of NaCl) by i.p. injections of 0.2 ml at 30 min before and 30 min, 1.5 h, 2.5 h, 3.5 h, 6 h, 9 h, and 12 h after CS challenge. NTM injections continued every 6 h until 54 h, then every 12 h until sacrifice at 168 h (7 days post-CS). Antibiotic therapy with meropenem (25 mg/kg administered s.c.) was begun at 12 h post-CS and continued every 12 h until sacrifice. Blood samples (~40 μL) were collected from the saphenous vein in EDTA before and at 2 h, 6 h, 12 h and 24 h post-CS challenge. Plasma was separated by centrifugation and stored at -80°C. Mice were closely monitored for survival throughout the experimental period.

For analyses of blood cell populations, bacterial colonization of blood and organs, and SRTFs in liver nuclear extracts, CS was prepared and administered as described above at a dose of 1.8 mg CS/g body weight. Control mice were given a sham dose of 5% dextrose alone adjusted to their body weight. CS groups were treated with 7 i.p. injections of cSN50.1 or vehicle prepared as described above. Mice were euthanized by isoflurane inhalation 12 h after CS/sham injection. Whole blood was collected in EDTA from the retro-orbital plexus. Lung, spleen and liver were harvested, snap-frozen in liquid nitrogen, and stored at -80°C, or for bacterial counts, dipped briefly in 70% ethanol then put on ice in 0.5 ml sterile PBS. One liver lobe was fixed in 10% formalin for histological analysis. Liver was perfused with sterile PBS before transfer to liquid nitrogen or formalin. Blood cell populations were determined in freshly collected whole blood by automated complete blood count in the Translational Pathology Shared Resource at Vanderbilt University.

### Nuclear extract preparation and immunoblotting

Nuclear extracts were prepared from frozen livers as previously described [19]. Briefly, liver pieces were disrupted in a Dounce hand homogenizer on ice without NP-40 and nuclei pelleted at 4000 x *g* for 1 minute before extract preparation. Extracts from 5 mice in each group (sham-infected, CS-infected + vehicle and CS-infected + cSN50.1) were analyzed for nuclear import of SRTFs by quantitative immunoblotting using polyclonal goat anti-cFos (Santa Cruz), monoclonal mouse anti- Y701phophorylated STAT1 (Becton-Dickinson) and polyclonal rabbit anti-NF-κBp50 and anti-NF-κB p65 (Abcam) on a Licor Odyssey Infrared Imaging System. Mouse monoclonal anti-TATA binding protein (TBP, Abcam) was used to measure TBP as a nuclear loading control for normalization.

### Bacterial cultures

Whole blood (50 μl) was diluted 1:1 with sterile PBS. Left lung and whole spleen were homogenized in 0.5 ml sterile PBS, and serial dilutions made in sterile PBS. Samples were plated on Tryptic Soy Agar + 5% sheep’s blood. Colonies were counted after overnight incubation at 37°C and results reported in CFU per milliliter of blood, or organ homogenate.

### Cytokine/chemokine assays

A cytometric bead array (BD BioSciences) was used to measure IL-6, IL-10, MCP-1, TNFα, and soluble E- and L-selectins in murine blood plasma following the manufacturer’s protocol and analyzed in the Flow Cytometry Core at Vanderbilt University as previously described [22]. IFN-γ and Soluble P-selectin were measured in murine blood plasma by ELISA (ThermoFisher Scientific and R&D Systems, respectively).

### Histological analyses

Tissues collected for microscopic analysis were fixed overnight in formalin then processed routinely, embedded in paraffin, sectioned at 5 microns and mounted on charged slides. Slides for pathology evaluation were stained with PAS or immunostained for neutrophils in the Translational Pathology Shared Resource at Vanderbilt University. Immunohistochemistry for neutrophils was performed on the Leica Bond Max (Leica Biosystems Inc. Buffalo Grove, IL) using Epitope Retrieval 2 solution for 20 minutes. Slides were incubated with anti-Neutrophil Marker (Cat. ab2557, Abcam, Cambridge, MA) for one hour at a 1:2000 dilution and then incubated in a rabbit anti-rat secondary (BA-4001, Vector Laboratories, Inc.) for 15 minutes at a 1:200 dilution. The Bond Polymer Refine Detection system was used for visualization. Immunostained slides were imaged at 20X magnification to a resolution of 0.5 μm/pixel using a high throughput Leica SCN400 Slide Scanner automated digital image system from Leica Microsystems. Upper and lower thresholds for color, saturation, intensity, size, roundness, and axis length were set for both blue Hematoxylin staining of nuclei and for brown DAB reaction products. Thus, brown (DAB) positive cells can be distinguished from blue (Hematoxylin only) negative cells. The percentage of positive brown (DAB-positive) stained cells was calculated as a total analyzed number of positive cells divided by total number cells in the section of liver. Whole slide imaging and quantification of immunostaining were performed in the Digital Histology Shared Resource at Vanderbilt University Medical Center.

### Statistics

GraphPad Prism software was used for statistical analyses. Blood cell counts, bacterial counts in blood, lung homogenates and liver homogenates, neutrophil immunostaining, plasma levels of sP-selectin at 24h and mean time to death were compared using the non-parametric Mann-Whitney *U* test. Cytokine, chemokine, sL-selectin and sE selectinlevels in plasma collected from the same animals at different time points were evaluated by repeated measures two-way ANOVA with Sidak’s post-test. SRTFs in nuclear extracts were analyzed by *t* test. Survival data were plotted as Kaplan-Meier survival curves and analyzed by the log-rank test. Data are presented as the means ± SEM and *p* values of < 0.05 were considered significant.

### Study approval

Animals were housed in groups of five in the animal care facility of Vanderbilt University in a 12 hour light/dark cycle. Regular rodent chow and water were provided *ad libitum*. Mice used in all experiments were 8–10 weeks of age. All animal handling and experimental procedures were performed in strict accordance with the recommendations in the Guide for the Care and Use of Laboratory Animals of the National Institutes of Health. The protocol was approved by the Vanderbilt University Animal Care and Use Program (Permit Number: A3227-01), which has been accredited by the American Association of Accreditation of Laboratory Animal Care International (file #000020). Frequent monitoring was employed to minimize animal suffering as much as possible. Analgesics could not be used in these studies as they modulate the inflammatory markers measured and interfere with experimental outcomes. For survival studies, mice were monitored at least every 6 hours for the first 54 hours after CS injection, then every 12 hours for an additional 5 days. The health condition of animals was scored at each time point on a scale from 1 (moribund) to 5 (healthy and active) based on the severity of symptoms such as hunched posture, reduced mobility, piloerection, tremor, hypothermia and paralysis. Mice with a score of 2 were checked more frequently throughout the 7 day study period, and some improved from this point. Mice with a score of 1 (inability to ambulate and/or severe respiratory distress) were euthanized immediately by isoflurane inhalation followed by cervical dislocation. There were no unexpected deaths, and all surviving mice were euthanized at the end of the study period by isoflurane inhalation followed by cervical dislocation.

## Supporting information

S1 Fig Parameters of the CBC shown in Fig 4B that are not changed by infection or NTM treatment (n = 5 mice/group). Bars represent median values from 5 mice/group. No significant differences were determined by Mann-Whitney test.
(PDF)

S2 Fig Full unedited SDS PAGE gels used for preparation of Fig 2A. (PDF)
