# Supplementary material for: Survival, bacterial clearance and thrombocytopenia are improved in polymicrobial sepsis by targeting nuclear transport shuttles
Source: PLoS One. 2017 Jun 19;12(6):e0179468. doi: 10.1371/journal.pone.0179468 (PMC5476269; doi:10.1371/journal.pone.0179468)
Supplement: S1 Fig — Bars represent median values from 5 mice/group. No significant differences were determined by Mann-Whitney test. (PDF) [file pone.0179468.s001.pdf]

Supporting Figure 1

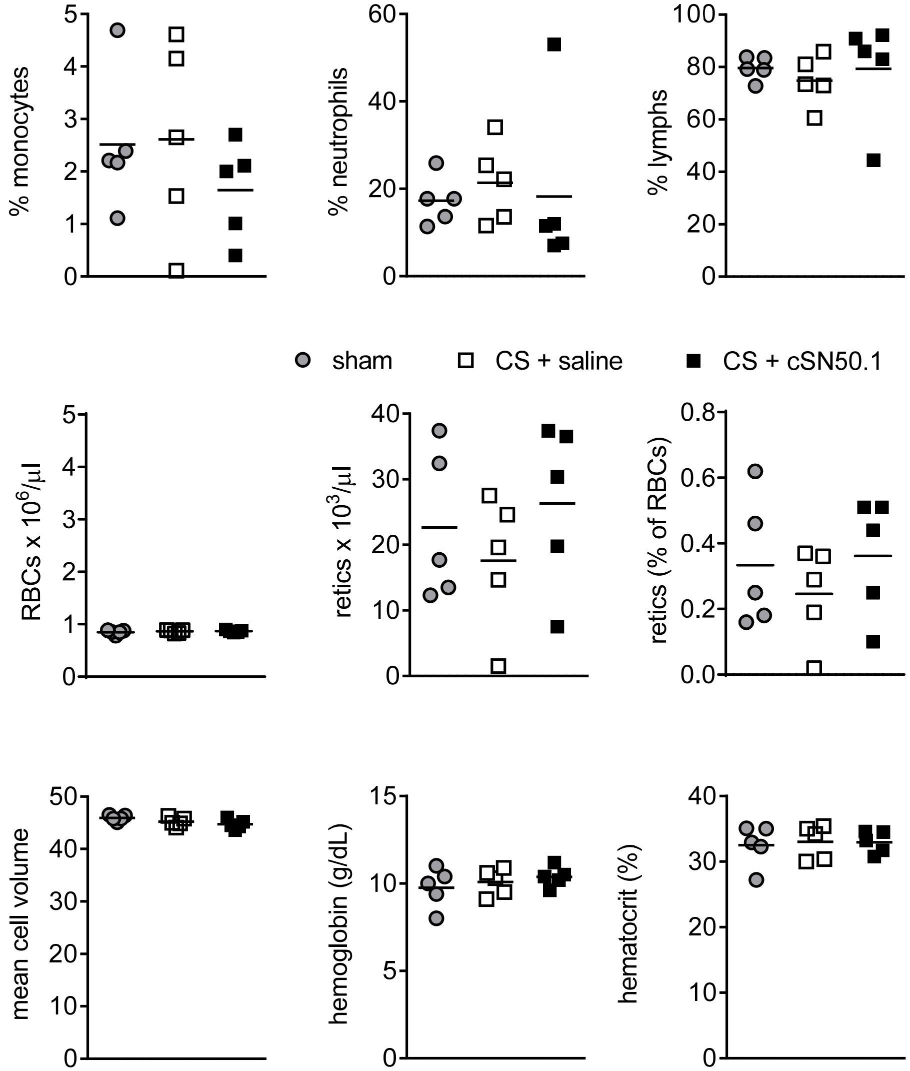

S1 Fig. Parameters of the CBC shown in Figure 4B that are not changed by infection or NTM treatment (*n*=5 mice/group). Bars represent mean values from 5 mice/group. No significant differences were determined by Mann-Whitney test.
