# Supplementary material for: Survival, bacterial clearance and thrombocytopenia are improved in polymicrobial sepsis by targeting nuclear transport shuttles
Source: PLoS One. 2017 Jun 19;12(6):e0179468. doi: 10.1371/journal.pone.0179468 (PMC5476269; doi:10.1371/journal.pone.0179468)
Supplement: S2 Fig — (PDF) [file pone.0179468.s002.pdf]

Supporting Figure 2

Full unedited gel #1 for Fig 2: blot cut horizontally between MW 50 and MW 70 (arrow)

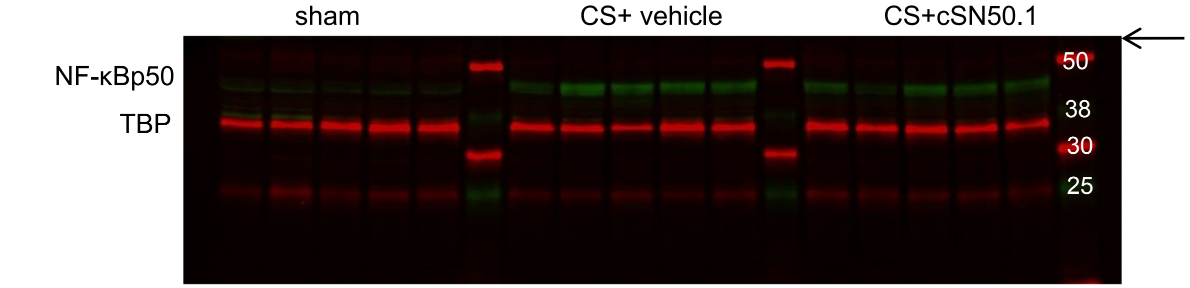

TOP: not shown in Fig 2
BOTTOM:
800 channel (green)
NF-κBp50 MW 50 kD
700 channel (red)
TBP MW 35 kD
NOTE: The 700 IRD anti-mouse secondary antibody used to detect mouse anti-TBP also detects the heavy and light chains of antibodies present in mouse tissue (red bands at ~50 kD and ~25 kD).

Full unedited gel #2 for Fig 2: blot cut horizontally at MW 70 and MW 38 (arrows)

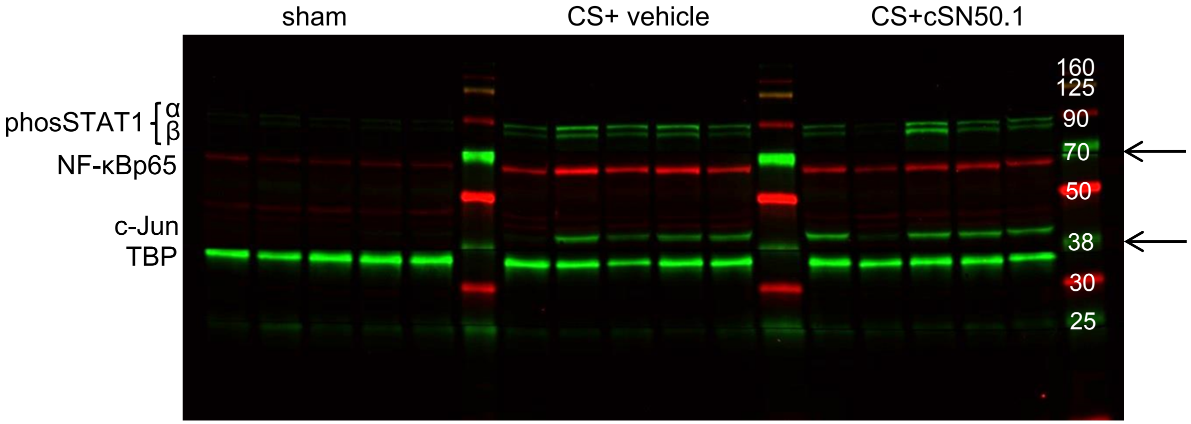

TOP:
800 channel (green)
Phospho-STAT1 (pY701):
Phospho-STAT1α MW 91
Phospho-STAT1β MW 84
No 700 channel (red)
MIDDLE:
700 channel (red)
NF-κBp65 MW 65 kD
800 channel (green)
C-Jun MW 43/48 (not shown in Fig 2)
BOTTOM:
800 channel (green)
TBP MW 35 kD
No 700 channel (red)
NOTE: The 800 IRD anti-mouse secondary antibody used to detect mouse anti-TBP also detects the light chain of antibodies present in mouse tissue (green band at ~25 kD).

Full unedited gel #3 for Fig 2: blot cut horizontally at MW 70 (arrow)

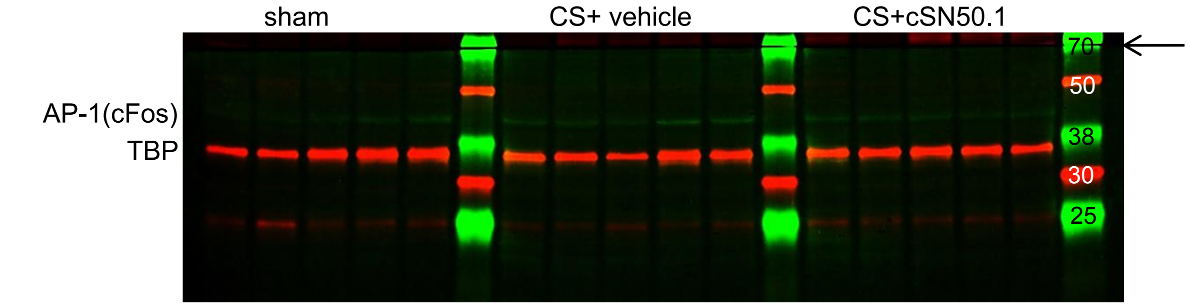

TOP: not shown in Fig 2
BOTTOM:
800 channel (green)
AP-1 cFos MW 40-60 kD
700 channel (red)
TBP MW 35 kD
NOTE: The 700 IRD anti-mouse secondary antibody used to detect mouse anti-TBP also detects the heavy and light chains of antibodies present in mouse tissue
(red bands at ~50 kD and ~25 kD).
